# Complex chromosome rearrangement in a child with microcephaly, dysmorphic facial features and mosaicism for a terminal deletion del(18)(q21.32-qter) investigated by FISH and array-CGH: Case report

**DOI:** 10.1186/1755-8166-1-24

**Published:** 2008-11-11

**Authors:** Emmanouil Manolakos, Nadezda Kosyakova, Loreta Thomaidis, Rozita Neroutsou, Anja Weise, Markos Mihalatos, Sandro Orru, Haris Kokotas, George Kitsos, Thomas Liehr, Michael B Petersen

**Affiliations:** 1 Bioiatriki SA, Athens, Greece; 2 Institute of Human Genetics and Anthropology, Jena, Germany; 3 1st Department of Pediatrics, University of Athens, "Aghia Sophia" Children's Hospital, Athens, Greece; 4 Genomedica SA, Piraeus, Greece; 5 Department of Medical Genetics, University of Cagliari, Cagliari, Italy; 6 Department of Genetics, Institute of Child Health, "Aghia Sophia" Children's Hospital, Athens, Greece; 7 Department of Ophthalmology, University of Ioannina, Ioannina, Greece

## Abstract

We report on a 7 years and 4 months old Greek boy with mild microcephaly and dysmorphic facial features. He was a sociable child with maxillary hypoplasia, epicanthal folds, upslanting palpebral fissures with long eyelashes, and hypertelorism. His ears were prominent and dysmorphic, he had a long philtrum and a high arched palate. His weight was 17 kg (25^th ^percentile) and his height 120 cm (50^th ^percentile). High resolution chromosome analysis identified in 50% of the cells a normal male karyotype, and in 50% of the cells one chromosome 18 showed a terminal deletion from 18q21.32. Molecular cytogenetic investigation confirmed a del(18)(q21.32-qter) in the one chromosome 18, but furthermore revealed the presence of a duplication in q21.2 in the other chromosome 18. The case is discussed concerning comparable previously reported cases and the possible mechanisms of formation.

## Background

Cases involving partial deletions or duplications of chromosome 18 are well documented in the literature. The 18q- syndrome constitutes one of the frequent autosomal deletion syndromes in man, with more than 100 patients reported [1]. The syndrome includes moderate intrauterine growth retardation, moderate mental retardation, and a specific pattern of dysmorphisms and anomalies [1]. Mosaicism for a deleted chromosome 18 has been described in a few patients with mostly the full clinical picture of the 18q- syndrome. Here, we report a patient with an unusual mosaic karyotype consisting of cells with normal karyotype and others with a terminal deletion of one chromosome 18 and the other chromosome 18 having an interstitial duplication.

## Methods

### Clinical findings

The patient, a 7-year-old boy, was the second child of unrelated, healthy parents. He was born with cesarean section after a full term pregnancy. His birth weight was 2,850 kg, length 45 cm and head circumference (HC) 32 cm. His perinatal period was uneventful. His developmental milestones were delayed as he sat independently at the age of 13 months and walked at the age of 27 months. His first words were spoken at the age of 2 years and 5 months.

He was a sociable child, with microcephaly (HC = 50.5 cm, 2^nd ^percentile), and dysmorphic facial features such as: maxillary hypoplasia, epicanthal folds, upslanting palpebral fissures, long eyelashes, and hypertelorism. His ears were prominent and dysmorphic and he had a high arched palate. His weight was 17 kg (25^th ^percentile) and his height 120 cm (50^th ^percentile).

His non-verbal skills were equivalent to a 4 years and 4 months level and his language skills were equivalent to a 30 months level. According to Griffiths Scales Bailey's Scales of Mental Development (2^nd ^Edition), his General Developmental Quotient (GDQ) was 52 with Performance DQ = 59 and Language DQ = 45. His behavior was normal for his developmental age. He was severely hypertonic but without asymmetry.

Heart auscultation was normal. Thyroid function and ultrasound were both normal. The bone age was increased (equivalent to 8 years and 3 months). Heart ultrasound (triplex) was normal. Brain MRI scan revealed a small dilatation of the lateral ventricles with one small focus of highly magnetic signal in the periventricular area. Kidney-liver-spleen ultrasound, EEG, visual and audiological examinations, and organic and amino acid levels were all normal.

### Cytogenetic and molecular cytogenetic analyses

Chromosomal preparation and GTG banding were done after lymphocyte culture according to standard procedures. A total of 100 cells were examined in the patient and his parents.

Fluorescence in situ hybridization (FISH) experiments were performed on metaphase spreads using the following commercially available probes (Abbott/Vysis): subtelomeric probe for 18qter and chromosome 18 centromeric probe (cep 18). Additionally, probes (BAC-PACs) for the chromosomal regions 18q21.2 (RP11-160B24) and 18q22.2 (RP11-704G7) were obtained from the Children's Hospital Oakland Research Institute (CHORI, Oakland, California). For the latter, probe labeling was done as previously reported [2]. FISH was done according to Liehr et al. [2]. Ten metaphase spreads including 5 normal and 5 aberrant cells were analyzed for each FISH experiment.

### Array-CGH

Genomic DNA was isolated from blood lymphocytes or from lymphoblastoid cell lines by routine procedures. Purification of genomic DNA, DNA labeling, hybridization of labeled DNA to a 4400 BAC clone micro-array (CytoChip™) and spot identification were performed according to the manufacturer's instructions (BlueGnome). In brief, 400 ng of genomic DNA was labeled by random priming with Cy3-dCTP or Cy5-dCTP (BlueGnome) and hybridized to 400 ng of sex-mismatched (female) reference DNA (Promega) labelled in the same way. Test and reference samples were mixed, co-precipitated, and resuspended in 25 μl of a hybridization solution containing 50% formamide, 10% dextran sulfate, 125 μg Cot-1 DNA (Roche), and 1.5 mg of herring sperm DNA (BlueGnome). A 22-h hybridization at 37°C was performed, followed by six post-hybridization wash cycles according to the manufacturer. Slides were dried by centrifugation and scanned using an InnoScan 700 Micro-Array Scanner (Innopsys). Spot identification and two-color fluorescence intensity measurements were obtained using the Mapix 2.9.5 software (Innopsys), and the data were entered into the BlueFuse Microarrays 3.5 software (BlueGnome) for subsequent analysis. Following normalization, the log_2 _transformed test-over-reference ratios were analyzed for loss and gain of genomic regions by a standard Hidden Markov Model [3].

## Results

### Cytogenetic and molecular cytogenetic analyses

**Figure 1:**
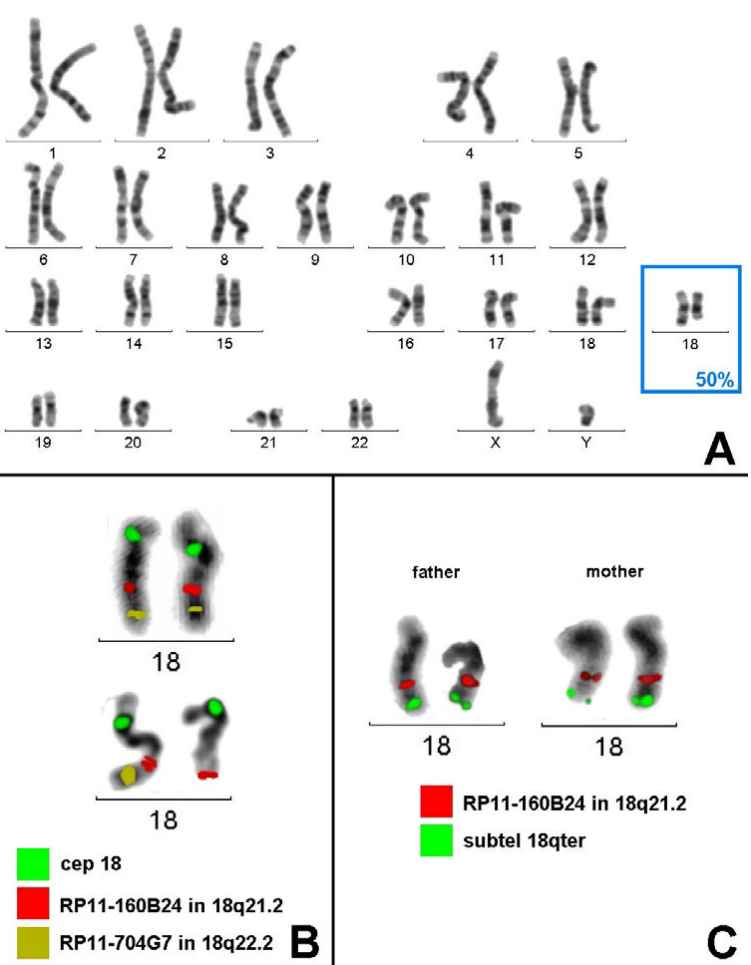
**Cytogenetic analysis of the proband and his parents**. A) GTG banding revealed a normal male karyotype in 50% of the cells. In the remainder, one deleted and one apparently normal chromosome 18 were present (partial karyotype in blue frame). B) FISH confirmed that both chromosomes 18 were normal in 50% of the cells. However, in the remaining cells both chromosomes 18 turned out to be rearranged. One had a terminal deletion del(18)(q21.32-qter), and the second showed a duplication/insertion of 18q21.2 material adjacent to the original location of the RP11-160B24 probe. C) No such duplication in 18q21.2 was present in any of the parents.

GTG banding revealed in 50% of the cells a normal male karyotype 46,XY; in the remaining cells a terminal deletion of the long arm of the one chromosome 18, 46,XY,del(18)(q21.32-qter), was observed (Fig. 1A). The chromosome abnormality occurred *de novo*, as both parents had a normal karyotype.

Molecular cytogenetic analyses were used to further characterize the chromosomal rearrangement. To determine if the telomere of the derivative chromosome 18 was present, FISH was carried out using a subtelomeric probe for the long arm. The subtelomeric probe was present on the normal chromosome 18, but absent on the deleted chromosome 18 (result not shown). To further characterize the del18qter, two BAC clones were selected: one on 18q21.2 (RP11-160B24) and one on 18q22.2 (RP11-704G7). FISH studies on metaphase chromosomes showed two signals in metaphases with the normal karyotype; in metaphases with the del(18qter), three signals for the 18q21.2 probe and one signal for the 18q22.2 probe were visible (Fig. 1B). In metaphases with the derivative chromosome del(18q), the apparently normal chromosome 18 displayed a previously cryptic duplication of the RP11-160B24 probe in the 18q21.2 region. This duplication/insertion occurred *de novo*, as no such rearrangement was present in the parents (Fig. 1C).

Based on these data, the karyotype was defined as: mos 46,XY.ish (wcp 18+/+, subtel18qter(Abott)+/+, cep18+/+, RP11-160B24+/+, RP11-704G7+/+ [50]/46XY,del(18q),dup(18q).ish (wcp18+/+, subtel18qter(Abott)+/+, cep 18+/+, RP11-160B24+/++, RP11-704G7-/+) [50].

### Array-CGH

**Figure 2:**
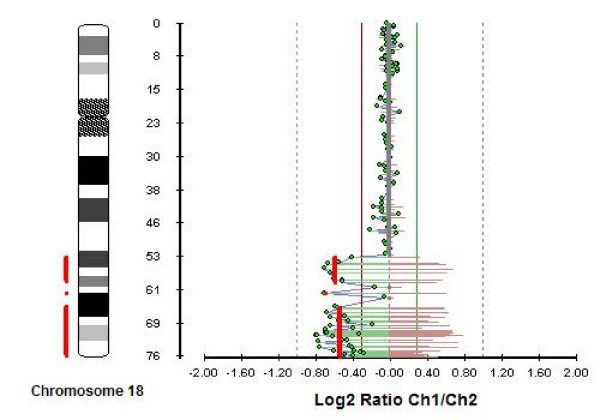
Array-CGH analysis of chromosome using a 4400 BAC clone micro-array (CytoChip™, BlueGnome).

The array-CGH analysis revealed two regions with deletion; one deletion encompassing about 5.66 Mb from 18q21.31 to 18q21.33 (region 53479498.5 – 59137161.5 on the chromosome) and one deletion encompassing about 11.16 Mb from 18q22.2 to 18q23 (region 64869098 – 76025498 on the chromosome) (Fig. 2). A third relatively smaller region between the two deletions mentioned above, was shown not to be deleted (about 2.2 Mb inside the 18q22.1 band).

## Discussion

The 18q- syndrome is one of the more frequent autosomal deletion syndromes in man, with more than 100 patients reported [1]. This deletion involves del(18)(q21-qter) with the majority of breakpoints in 18q21.2-q22.2 [4]. About 75% of patients have *de novo *deletions with the remainder having unbalanced translocations or inversions. Of the *de novo *cases, 85% are paternal in origin [5]. The degree of mental retardation, growth retardation and malformations does apparently not correlate with the breakpoint position [1]. Most patients with subband determination have the breakpoint at 18q21.3 and these patients rarely have congenital malformations.

The syndrome comprises moderate intrauterine growth retardation, facial dysmorphism, moderate mental retardation, and various anomalies [1]. The facial dysmorphism may include brachycephaly, midface hypoplasia, everted lower lip, narrow upper lip with absent philtrum, downturned corners of the mouth, depressed and wide nasal bridge, small teeth, deep-set eyes with downslanting palpebral fissures, epicanthus, strabismus, prominent anthelix and antitragus, and very narrow external ear canal [1]. The associated anomalies include kyphosis/scoliosis, vertebral anomalies, genital hypoplasia, proximally placed thumbs, tapering fingers, clinodactyly of fifth fingers, coxa valga, clubfeet, toe anomalies, dimples over joints, transverse palmar creases, congenital heart defects, hypospadias, cleft lip and palate, deafness, and several less frequent anomalies [6,1]. A recent study using high-resolution array-CGH in 29 patients with such deletions concluded that the critical region for the 'typical' 18q- phenotype is a region of 4.3 Mb located within 18q22.3-q23 [7].

The neuropsychiatric phenotype has been characterized in 27 patients with age range 2–47 years [8]. Intelligence ranged from borderline to severely deficient. Performance in specific neuropsychological functions was consistently in the moderately-to-severely impaired range. Behavioral problems were common and 6% of the patients had autism. There was no relationship between deletion size and any measure of cognition or behavior [8].

Mosaicism for a deleted chromosome 18 was repeatedly found in patients with mostly the full, and occasionally a diminished clinical picture of the 18q- syndrome. Partial monosomy and partial trisomy involving different segments of chromosome 18 in the same patient have been described in pericentric inversion recombinants [9]. The patient of an early report had a clinical diagnosis of 18q- syndrome and a recombinant chromosome 18 with deficiency of the distal one third of 18q and duplication of the terminal segment of 18p [9]. The mechanism here involves crossover within the formed inversion loop, so called 'aneusomie de recombinaison' [10]. A maternal paracentric 18q inversion gave rise to a recombinant del/dup18q in the twin offspring [11]. Molecular cytogenetic and molecular analyses suggested that the duplication-deletion chromosome resulted from breakage of a dicentric recombinant chromosome 18 [11]. The same mechanism of an intermediate dicentric chromosome undergoing breakage and giving rise to a deleted and an inv dup chromosome, has been demonstrated for mono- and dicentric 8p duplications [12].

A mosaic karyotype 46,XY,del(18)(q21.3q22.2)/47,XY,del(18)(q21.3q22.2)+r(18) was found in a mentally retarded male with a mild form of the 18q- syndrome [13].

A mentally retarded boy with 46,XY,del(18)(q22)/46,XY,psu idic(18)(q23) had clinical manifestations compatible with the 18q- syndrome [14]. A possible mechanism involving an early postzygotic U-type exchange of sister chromatids at 18q23 (yielding an unstable dicentric chromosome), breakage at 18q22 (resulting in the 18q- chromosome of one cell line), and inactivation of one centromere (giving a stable pseudoisodicentric chromosome 18 in the other cell line) was presented [14]. A mosaic isopseudodicentric chromosome 18q was detected in a girl with discrete features of trisomy 18 [15]. The 18q23 breakpoint was very distal as FISH with one of the most telomere located probes could not confirm monosomy of distal 18q23 [15]. A few cases of isopseudodicentric chromosome 18q have been reported and most of the cases are mosaic, although one non-mosaic case has been described recently [16]. In that case, the isopseudodicentric chromosome 18q was present in 100% of blood lymphocytes, and a terminal deletion 18q with identical 18q22.1 breakpoint as the i(18q), was detected in placental cell cultures [16]. The authors pointed out that the child represented the first non-mosaic case being viable, although with severe congenital malformations and neonatal death at 14 weeks of age [16].

Three cell line mosaicism including full trisomy 18, normal cells and the majority of cells with partial trisomy involving an extra chromosome 18 deleted at band q22, was described in a girl with cardiac and CNS anomalies, dysmorphic facial features, failure to thrive, and developmental delay [17]. Non-disjunction studies of trisomy 18 have demonstrated that an error in maternal meiosis II (MII) is the most frequent cause of non-disjunction leading to trisomy 18 [18], unlike human trisomies 13, 15, 16, and 21, which show a higher frequency of maternal meiosis I (MI) errors [19]. In the present case, microsatellite analysis indicated maternal meiotic origin of the extra chromosome 18 and paternal origin of the deleted chromosome 18 [17]. The authors suggested a trisomic conception followed by two early postzygotic (mitotic) errors as the simplest mode to explain the karyotype of this patient.

A baby girl with severe growth retardation, developmental delay, microcephaly, and multiple congenital anomalies, had a mosaic karyotype consisting of cells with an i(18q) and another cell line with a r(18q) [20]. The authors noticed that the isochromosome was probably formed by centromere misdivision with loss of the i(18p) chromosome. They further stated that the rearrangement had arisen before conception and suggested that the ring chromosome originated from the cells with an i(18q) chromosome by breakage in both long arms with subsequent reunion, a mechanism which had been previously described in ring chromosome 21 formation [21]. A recent study on ring chromosome formation using array-CGH showed not only the expected terminal deletion but also a contiguous duplication through a classical inv dup del rearrangement [22].

**Figure 3:**
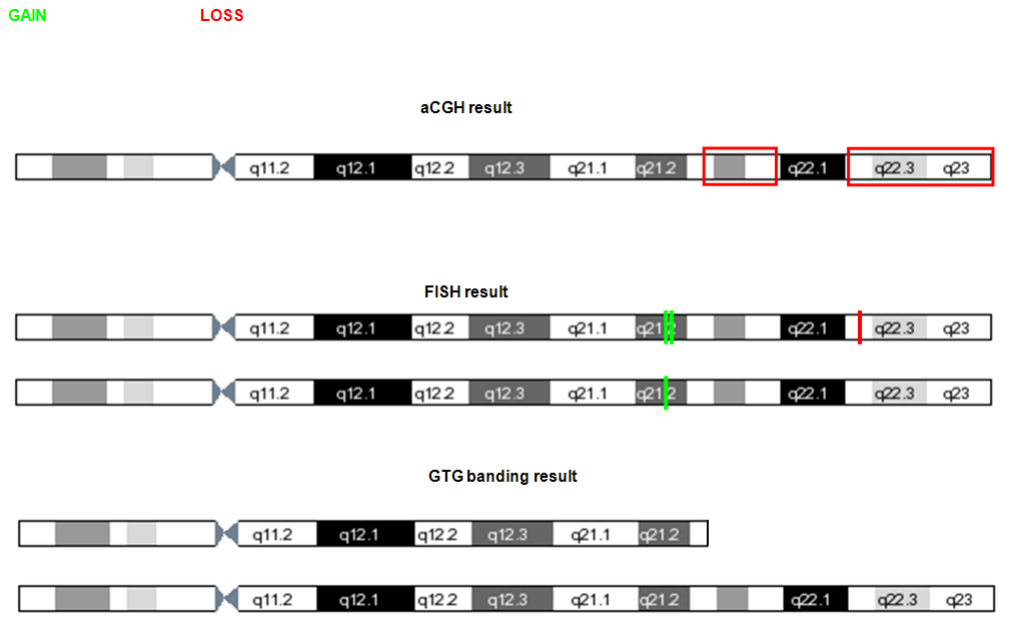
Ideogram of chromosome 18 presenting the results of GTG banding, FISH analysis and array-CGH in the proband.

In Fig. 3 we present a schematic drawing of the comprehensive results of GTG banding, FISH and array-CGH in the proband. The FISH results are in agreement with the cytogenetic findings, if the 18q21.2 duplication represented by BAC clone RP11-160B24 is small. The 18q deletion detected by array-CGH is in agreement with the size of the cytogenetic deletion, if we assume that 18q21.3-18qter is deleted on the one chromosome. The finding by array-CGH of a relatively small not-deleted region (about 2.2 Mb) within the deleted region is intriguing, and can be interpreted as a duplication located on the other chromosome. A 2.2 Mb duplication would not be visible by ordinary GTG banding, neither by FISH if probes are not used within the duplicated area. The array-CGH cannot discern whether the 2.2 Mb duplication is located on the chromosome with the terminal deletion or on the other chromosome with the duplication represented by BAC clone RP-160B24. The extent of the FISH duplication was not further investigated and was not picked up by array-CGH.

Low copy repeats (LCRs) spread over the human genome are stretches of duplicated DNA more than 1 kb in size. Non-allelic homologous recombination between highly similar LCRs has been implicated in numerous genomic disorders [23], and LCRs have also been characterized at breakpoints of both balanced and unbalanced chromosomal rearrangements [24]. It is interesting to note that in our patient, BAC clone RP11-160B24 showed a duplication in band 18q21.2, with the deletion breakpoint being located in adjacent band 18q21.31 (as determined by array-CGH). The use of the array-CGH technique has revealed the unbalanced or complex nature of a proportion of *de novo *apparently balanced translocations [25]. In 27 cases of *de novo *reciprocal translocations in patients with an abnormal phenotype, 11 (40%) were found to be unbalanced after array-CGH analysis [25]. Furthermore, 5 out of 27 (18%) of the reciprocal translocations were instead complex rearrangements with more than 3 breakpoints [25].

## Conclusion

It is important to keep in mind that, like in our case, an unexpected duplication in deletion cases might confuse the genotype-phenotype correlation, and array-CGH continues to be an important technique in characterizing chromosomal rearrangements, their mechanisms of formation, precise establishment of genotype-phenotype correlations, and genetic counseling.

## Consent

Written informed consent was obtained from the parents of the patient for publication of this case report and any accompanying images. A copy of the written consent is available for review by the Editor-in-Chief of this journal.

## Competing interests

The authors declare that they have no competing interests.

## Authors' contributions

EM performed the cytogenetic analysis, collected all data and drafted the manuscript, NK performed the molecular cytogenetic analysis, LT provided clinical data of the patient, RN performed the cytogenetic analysis, AW performed the molecular cytogenetic analysis, MM performed the array-CGH analysis, NP performed the array-CGH analysis, SO participated in the design and coordination of the study, HK participated in the coordination of the study and contributed to the completeness of the final manuscript, GK participated in the design and coordination of the study, TL performed the molecular cytogenetic analysis and contributed to the final version, and MBP coordinated the study and contributed to the final version of the manuscript.

All authors read and approved the final manuscript.
